# Supplementary material for: The Synthesis of a Covalent Organic Framework from Thiophene Armed Triazine and EDOT and Its Application as Anode Material in Lithium-Ion Battery
Source: Polymers (Basel). 2021 Sep 27;13(19):3300. doi: 10.3390/polym13193300 (PMC8512810; doi:10.3390/polym13193300)
Supplement: Supplementary file 1 [file polymers-13-03300-s001.zip › polymers-1315533-supplementary.pdf]

polymers

Supplementary Material: The Synthesis of a Covalent Organic Framework from Thiophene Armed Triazine and EDOT, and Its Composite with Carbon Used as Anode Material in Lithium-ion Battery

Shuang Chen, Shukun Wang, Xin Xue, Jinsheng Zhao, Hongmei Du

Materials

5,7-bis(trimethylstannyl)-2,3-dihydrothieno[3,4-b][1,4]dioxine (2SnEDOT) was purchased from SunaTech Inc (Suzhou, China). 5-bromothiophene-2-carbonitrile (BTCN) was purchased from Zhengzhou Alfa Chemical Co., Ltd (Zhengzhou,China). Bis(triphenylphosphine)palladium ($PdCl_2(PPh_3)_2$), Vulcan XC-72 carbon, Tetrabutylammonium hexafluorophosphate ($TBAPF_6$, 98%), acetonitrile (ACN), 1-methyl-2-pyrrolidinone (NMP, 99.9%), ethanol, acetone, toluene and trifluoromethanesulfonic acid (TfOH) were bought from Aladdin Co., LTD (Shanghai, China). All reactants are of analytical grade and used without further purification.

Characterization Techniques

The FT-IR measurements were recorded on a Nicolet Avatar 360 FT-IR spectrometer with KBr pellets. The UV-Vis absorption spectroscopy was recorded on Varian Carry 5000 spectrophotometer (Agilent Technologies Ltd, Mulgrave, Australia). The morphologies of the samples were observed by Hitachi Su-70 scanning electron microscopy (SEM, Hitachi Inc., Tokyo, Japan). The specific surface areas and porosity properties was examined by Nitrogen isotherm adsorption-desorption at 77.3 K using ASAP 2460-3 (Micromeritics) volumetric adsorption analyzer (Micromeritics,USA). X-ray photoelectron spectroscopy (XPS) was conducted with ESCALAB 250Xi spectrometer (Thermo Fisher Scientific, Waltham, MA, USA). X-ray diffraction (XRD) was carried out with the 2θ range from 5 to 80° using Kigaka D/max 2500 X-ray advance diffractometer with a Cu-Ka radiation (Rigaku Corporation,Tokyo, Japan), and a step scan mode was adopted with a scanning step of 0.02°. The thermogravimetric analysis of the samples were conducted on a Netzsch STA449C TG/DSC thermal analyzer (TGA, TG, NETZSCH Scientific Instruments Trading Ltd., Germany) under nitrogen atmosphere between 20 °C and 800 °C.

Electrochemical Measurements

The electrochemical performances of the anode composites were tested with CR2032-type coin cells. A mixture is obtained by mixing the active material, acetylene black and polyvinylidene fluoride (PVDF) at a mass ratio of 6: 2.5: 1.5. The moderate amount of NMP was added to the mixture, be grinded thoroughly to form a homogeneous slurry, and the slurry was then coated on copper foils. The coated copper foil was dried at 60 °C for 24 h, and then be cut into slices as the working electrode (anode for LIBs). The slices are further dried in vacuum drier at 120 °C for 8 hours, the constant weight of a slice was about 9 mg with the diameter of 12 mm. Then the as-prepared electrode was paired with Li foil as the counter electrode and the half battery was assembled in an argon-filled glove box. The electrolyte used was the solution of 1 M $LiPF_6$ dissolved in ethylene carbonate (EC) and dimethyl carbonate (DMC) (1:1 v/v) mixture. Galvanostatic charge-discharge experiments and rate capability were carried out on a land battery testing system (Land CT2001A, Wuhan, China) at room temperature. The same configured cells were also subject to cyclic voltammetry (CV) and electrochemical impedance spectroscopy (EIS) measurements on a potentiostat (PGSTAT 302N, Metrohm) (Metrohm, Herisau, Switzerland).

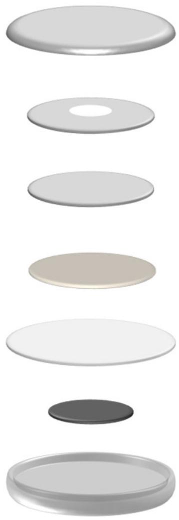

Figure S1. The schematic diagram of the coin-type battery.

Table S1. The performance of some CTFs@C composites as anode materials for LIBs.

Name	C_n (mA h g^{-1}) cycle number	Electrochemical window (V)	Electrode composition (AM:CA:B)[wt%]	Addition	Binder	Ref
PTT-1@C	495, 300	0-3 V	6:2.5:1.5	AB	PVFD	21
PTT-2@C	671, 300	0-3 V	6:2.5:1.5	AB	PVDF	21
PTT-3@C	707, 300	0-3 V	6:2.5:1.5	AB	PVDF	21
PTT-4@C	772, 300	0-3 V	6:2.5:1.5	AB	PVDF	21
PTT-O/C	645, 300	0-3 V	6:2.5:1.5	AB	PVDF	this work

C_n (mA h g^{-1}): the specific capacity at determined cycle; AM, active material; CA, carbon additive; B, binder. AB, acetylene black; PVDF, poly(vinylideneuoride).
